# Neutrophil phenotypes and functions in cancer: A consensus statement

**DOI:** 10.1084/jem.20220011

**Published:** 2022-05-06

**Authors:** Daniela F. Quail, Borko Amulic, Monowar Aziz, Betsy J. Barnes, Evgeniy Eruslanov, Zvi G. Fridlender, Helen S. Goodridge, Zvi Granot, Andrés Hidalgo, Anna Huttenlocher, Mariana J. Kaplan, Ilaria Malanchi, Taha Merghoub, Etienne Meylan, Vivek Mittal, Mikael J. Pittet, Andrea Rubio-Ponce, Irina A. Udalova, Timo K. van den Berg, Denisa D. Wagner, Ping Wang, Arturo Zychlinsky, Karin E. de Visser, Mikala Egeblad, Paul Kubes

**Affiliations:** 1 Rosalind and Morris Goodman Cancer Institute, Department of Physiology, McGill University, Montreal, Quebec, Canada; 2 Cellular and Molecular Medicine, University of Bristol, Bristol, UK; 3 Center for Immunology and Inflammation, Feinstein Institutes for Medical Research, Manhasset, NY; 4 Center for Autoimmune, Musculoskeletal and Hematopoietic Diseases, Feinstein Institutes for Medical Research, Manhasset, NY; 5 Departments of Molecular Medicine and Pediatrics, Donald and Barbara Zucker School of Medicine at Hofstra/Northwell, Hempstead, NY; 6 Division of Thoracic Surgery, Department of Surgery, Perelman School of Medicine at the University of Pennsylvania, Philadelphia, PA; 7 Hadassah Medical Center, Institute of Pulmonary Medicine, Faculty of Medicine, Hebrew University of Jerusalem, Jerusalem, Israel; 8 Board of Governors Regenerative Medicine Institute and Research Division of Immunology, Department of Biomedical Sciences, Cedars-Sinai Medical Center, Los Angeles, CA; 9 Department of Developmental Biology and Cancer Research, Hebrew University of Jerusalem, Jerusalem, Israel; 10 Vascular Biology and Therapeutics Program and Department of Immunobiology, Yale University School of Medicine, New Haven, CT; 11 Area of Cell and Developmental Biology, Centro Nacional de Investigaciones Cardiovasculares Carlos III, Madrid, Spain; 12 Department of Medical Microbiology and Immunology, University of Wisconsin-Madison, Madison, WI; 13 Department of Pediatrics, University of Wisconsin-Madison, Madison, WI; 14 Systemic Autoimmunity Branch, Intramural Research Program, National Institute of Arthritis and Musculoskeletal and Skin Diseases, National Institutes of Health, Bethesda, MD; 15 Tumour-Host Interaction Laboratory, The Francis Crick Institute, London, UK; 16 Ludwig Collaborative and Swim Across America Laboratory, Memorial Sloan Kettering Cancer Center, New York, NY; 17 Parker Institute for Cancer Immunotherapy, Memorial Sloan Kettering Cancer Center, New York, NY; 18 Department of Medicine, Memorial Sloan Kettering Cancer Center, New York, NY; 19 Weill Cornell Medical College, New York, NY; 20 Lung Cancer and Immuno-Oncology Laboratory, Bordet Cancer Research Laboratories, Institut Jules Bordet, Université Libre de Bruxelles, Anderlecht, Belgium; 21 Laboratory of Immunobiology, Université Libre de Bruxelles, Gosselies, Belgium; 22 Department of Cardiothoracic Surgery, Neuberger Berman Foundation Lung Cancer Research Center, Weill Cornell Medicine, New York, NY; 23 Department of Cell and Developmental Biology, Weill Cornell Medicine, New York, NY; 24 Department of Pathology and Immunology, University of Geneva, Geneva, Switzerland; 25 Ludwig Institute for Cancer Research, Lausanne Branch, Lausanne, Switzerland; 26 Department of Oncology, Geneva University Hospitals, Geneva, Switzerland; 27 AGORA Cancer Research Center, Lausanne, Switzerland; 28 University of Oxford, Kennedy Institute of Rheumatology, Oxford, UK; 29 Laboratory of Immunotherapy, Sanquin Research, Amsterdam, Netherlands; 30 Department of Molecular Cell Biology and Immunology, Amsterdam University Medical Center, Amsterdam, Netherlands; 31 Program in Cellular and Molecular Medicine, Division of Hematology/Oncology, Boston Children’s Hospital and Harvard Medical School, Boston, MA; 32 Department of Cellular Microbiology, Max Planck Institute for Infection Biology, Berlin, Germany; 33 Division of Tumour Biology and Immunology, Oncode Institute, Netherlands Cancer Institute, Amsterdam, Netherlands; 34 Department of Immunohematology and Blood Transfusion, Leiden University Medical Centre, Leiden, Netherlands; 35 Cold Spring Harbor Laboratory, Cold Spring Harbor, NY; 36 Department of Pharmacology and Physiology, Cumming School of Medicine, University of Calgary, Calgary, Alberta, Canada; 37 Banbury Center meeting organizers, Diverse Functions of Neutrophils in Cancer, Cold Spring Harbor Laboratory, New York, NY; 38 Department of Microbiology, Immunology & Infectious Diseases, Cumming School of Medicine, University of Calgary, Calgary, Alberta, Canada; 39 Calvin, Phoebe and Joan Snyder Institute for Chronic Diseases, University of Calgary, Calgary, Alberta, Canada

## Abstract

Neutrophils are the first responders to infection and inflammation and are thus a critical component of innate immune defense. Understanding the behavior of neutrophils as they act within various inflammatory contexts has provided insights into their role in sterile and infectious diseases; however, the field of neutrophils in cancer is comparatively young. Here, we summarize key concepts and current knowledge gaps related to the diverse roles of neutrophils throughout cancer progression. We discuss sources of neutrophil heterogeneity in cancer and provide recommendations on nomenclature for neutrophil states that are distinct in maturation and activation. We address discrepancies in the literature that highlight a need for technical standards that ought to be considered between laboratories. Finally, we review emerging questions in neutrophil biology and innate immunity in cancer. Overall, we emphasize that neutrophils are a more diverse population than previously appreciated and that their role in cancer may present novel unexplored opportunities to treat cancer.

## Teaser

There is a growing appreciation for the vastness of neutrophil functional states in cancer. Quail et al. provide a consensus statement on mechanisms governing neutrophil heterogeneity in the context of malignancy and discuss controversies and solutions in neutrophil research.

## Introduction

Neutrophils are polymorphonuclear granulocytes of the innate immune system that are the first line of defense to fight infection and maintain tissue homeostasis. They differentiate within the bone marrow (BM) to yield short-lived cytotoxic cells whose ebbs and flows in the vasculature and tissues are diurnally regulated. Neutrophils are highly abundant in circulation, accounting for up to ∼70% of all peripheral leukocytes in humans and ∼10–20% in mice (Mestas and Hughes, 2004). Rough estimates suggest humans produce ∼1 billion neutrophils daily per kilogram of body weight at steady state, and this may extend to 10 billion under inflammatory conditions (Ley et al., 2018). Neutrophils contain granules with an arsenal of cytotoxic factors, such as antimicrobial compounds, serine proteases, lysozyme, defensins, metalloproteases, and enzymes that mediate oxidative burst (Amulic et al., 2012). Their principal innate functions include degranulation, phagocytosis, and release of neutrophil extracellular traps (NETs; expelled DNA webs decorated with microbicidal proteins such as myeloperoxidase, elastase, and defensins). In addition, emerging studies indicate that some neutrophils can present antigens, co-regulate T cell responses, and kill in an antibody-dependent manner (Mantovani et al., 2011; Matlung et al., 2018). Usually, neutrophil cytotoxicity is beneficial and necessary to fight off infection; however, under chronic conditions, it can cause collateral tissue damage, particularly within highly vascularized tissues (Adrover et al., 2020; Adrover et al., 2019; Blanco et al., 2021; Clark et al., 2007).

Neutrophils are recruited to sites of sterile injury in large numbers, challenging the notion that these cells are exclusively antimicrobial and raising the possibility that they actively orchestrate tissue repair (Peiseler and Kubes, 2019; Phillipson and Kubes, 2011). This includes cancer, where various pro-tumorigenic functions of neutrophils have been described. Notably, neutrophils promote almost every aspect of cancer progression, such as primary tumor growth and metastasis (Coffelt et al., 2015; Cools-Lartigue et al., 2013; Demers et al., 2016; El Rayes et al., 2015; Engblom et al., 2017; Gershkovitz et al., 2018a; Park et al., 2016; Quail et al., 2017; Yang et al., 2020), cancer stem cell maintenance (Wculek and Malanchi, 2015), exit from dormancy and cell cycle progression (Albrengues et al., 2018; Houghton et al., 2010; Szczerba et al., 2019), impaired immunosurveillance (Casbon et al., 2015; Shaul et al., 2021; Spiegel et al., 2016; Veglia et al., 2019; Yajuk et al., 2021), and therapeutic resistance (Salvagno et al., 2019; Siwicki et al., 2021; Wisdom et al., 2019). Nevertheless, other studies have found that neutrophils can have an anti-tumorigenic role, including cytotoxicity against tumor cells (Bouti et al., 2021; Cui et al., 2021; Gershkovitz et al., 2018a; Gershkovitz et al., 2018b; Hirschhorn-Cymerman et al., 2020; Martinez Sanz et al., 2021; Martínez-Sanz et al., 2021; Matlung et al., 2018) and enhanced tumor cell clearance (Blaisdell et al., 2015; Eruslanov et al., 2014; Singhal et al., 2016), particularly in early-stage disease. Given the discordant mechanisms by which neutrophils can influence cancer, it is apparent that we lack a fundamental understanding of how neutrophil biology shifts in the context of malignancy.

This consensus statement follows a meeting at the Banbury Center at Cold Spring Harbor Laboratory focused on new and emerging concepts in the field of neutrophils in cancer. Here we summarize and expand on those discussions by reviewing current literature on neutrophil heterogeneity, discrepancies in the field, and open questions requiring further investigation.

## The unheeded complexity of neutrophil heterogeneity in cancer

Classical views of neutrophils in cancer have adopted a binary classification system that compartmentalizes neutrophils as either pro- or anti-tumorigenic. For many years, this has served as a satisfactory working hypothesis; however, with emerging research, it is clear that this polarized paradigm is inadequate. Akin to our evolving understanding of macrophage diversity (Ginhoux et al., 2016), recent research has identified diverse neutrophil states with widespread functionality. The effects of neutrophils on tumor biology have been covered by several excellent reviews (Coffelt et al., 2016; Engblom et al., 2016; Giese et al., 2019; Granot, 2019; Hedrick and Malanchi, 2022; Jaillon et al., 2020; Nicolas-Avila et al., 2017; Shaul and Fridlender, 2019). Therefore, we focus our discussion on the reciprocal effect, i.e., how the tumor and host environments regulate neutrophil heterogeneity (quantity and quality) to yield highly diverse cellular states with broad functionality.

### Neutrophil phenotypes driven by the tumor

#### Tumor regulation of neutrophil quantity

Tumor-induced neutrophilia requires signals to expand neutrophil progenitor pools and mediate chemotaxis. Two CSFs critical for granulopoiesis are granulocyte (G)-CSF (CSF-3) and GM-CSF (CSF-2; Hamilton and Achuthan, 2013). Early studies identified that highly metastatic tumors were capable of secreting G-CSF to stimulate the accumulation of neutrophils to promote metastasis (Kowanetz et al., 2010). The effect of tumor- or stroma-derived G-CSF on Ly6G^+^ or Gr1^+/hi^ granulocytes has since been confirmed by many additional groups (Casbon et al., 2015; Coffelt et al., 2015; Hsu et al., 2019; Strauss et al., 2015; Wculek and Malanchi, 2015; Welte et al., 2016), and it is now known that mechanistically, tumor-derived G-CSF skews hematopoiesis within BM toward the myeloid lineage resulting in elevated systemic frequencies of both immature and mature neutrophils with immunosuppressive properties in mice (Casbon et al., 2015). G-CSF production by tumors can be regulated upstream by IL-23 and IL-17 supplied by phagocytes and T cells, respectively (Coffelt et al., 2015; Smith et al., 2007; Stark et al., 2005). Additional studies have suggested a similar role for tumor- or stroma-derived GM-CSF in promoting the expansion of neutrophils and their progenitors in association with cancer progression (Bayne et al., 2012; Bronte et al., 1999; Bronte et al., 2003; Kohanbash et al., 2013; Wu et al., 2014). The central role of these CSFs in tumor-associated inflammation mirrors that of emergency granulopoiesis, a survival mechanism to systemically control disseminated infections when the immune system becomes maximally challenged (Manz and Boettcher, 2014). As such, questions have emerged over the use of G/GM-CSF in cancer patients following chemotherapy-induced myelosuppression. This is now being explored in retrospective studies, in which, fortunately, G-CSF does not appear to exacerbate brain metastasis in patients with de novo stage IV breast cancer (Fujii et al., 2021); however, these analyses need to be expanded to additional cancer contexts.

Activation of the chemokine receptor CXCR2 is a key event for neutrophil mobilization from BM (Ley et al., 2018), whereas BM retention is regulated by CXCR4 (Adrover et al., 2019; Eash et al., 2010; Martin et al., 2003). The coordination of CXCR2 and CXCR4 is regulated diurnally and underlies circadian pattern of neutrophil flux in steady state (Adrover et al., 2019; Casanova-Acebes et al., 2013). In human cancer, the CXCR2 ligand CXCL8 (IL-8) is abundantly secreted by various tumor types (Sanmamed et al., 2014) and is sufficient to regulate neutrophil recruitment and NETosis (Alfaro et al., 2016; Nie et al., 2019). Consistently, CXCR2 inhibitors can reduce tumor-associated NETs in models of melanoma, breast cancer, and colorectal cancer (Park et al., 2016; Park et al., 2015; Teijeira et al., 2020). Neutrophil CXCR2 signaling has been linked to adaptive immune responses by CD8^+^ T cells, and there is interest in combining CXCR2 inhibitors with immunotherapy. For example, in models of colitis-induced tumorigenesis, CXCR2 signaling within Ly6G^+^ myeloid cells suppresses tumoricidal functions of CD8^+^ T cells, as assessed by CD107a, IFNɣ, Prf1, and Gzmb (Katoh et al., 2013). In murine oral, renal, and pancreatic tumor models, CXCR2 inhibition results in neutrophil depletion coinciding with increased survival, T cell infiltration, and immunotherapy response (Chao et al., 2016; Najjar et al., 2017; Steele et al., 2016; Sun et al., 2019). In preclinical models of childhood cancers such as rhabdomyosarcoma, CXCR2^+^Ly6G^+^ cells mediate local immunosuppression, while CXCR2 inhibition improves immune checkpoint blockade efficacy (Highfill et al., 2014). Similarly, retrospective studies have shown that serum IL-8 is an independent biomarker of reduced efficacy of immune checkpoint inhibitors in patients, and coincides with increased neutrophils within tumors (Schalper et al., 2020; Yuen et al., 2020). Preclinical studies have implicated other CXCR2 ligands in neutrophil recruitment to tumors (Liu et al., 2016), and it is possible that these may serve as additional biomarkers in patients. In the era of cancer immunotherapy, these findings highlight the importance of understanding neutrophil dynamics as they relate to anti-tumor T cell responses.

In addition to specific tumor-supplied factors, genetic events within cancer cells underlie a broader shift in the tumor secretory profile with compounded effects on microenvironmental composition (Duits and de Visser, 2021). One such event is *Tp53* loss, which has pronounced effects on the tumor myeloid landscape. In breast cancer, loss of *Tp53* upregulates the secretion of WNT ligands, which stimulate macrophages to produce IL-1β, mediate neutrophilic inflammation, and potentiate metastatic progression (Wellenstein et al., 2019). In lung cancer, *Tp53* loss and *Kras* activation lead to elevated levels of the receptor for advanced glycation end products (RAGE) within blood, which educates distant osteoblasts to stimulate the expansion of long-lived pro-tumorigenic SiglecF^hi^ neutrophils (in contrast, SiglecF^lo^ neutrophils are enriched in healthy lung; Engblom et al., 2017; Pfirschke et al., 2020). In *Kras*-driven pancreatic cancer, loss of *Tp53* stimulates the production of myeloid chemokines, including CXCR2/3 ligands, resulting in the recruitment of immunosuppressive CD11b^+^ myeloid cells (Blagih et al., 2020). In prostate cancer, the combination of *Pten* and *Tp53* loss enhances CXCL17 secretion and recruitment of CD11b^+^Gr1^+^ immunosuppressive neutrophils (Bezzi et al., 2018). Combined loss of *Pten* and *Tp53* is also linked to CD11b^+^Gr1^+^ myeloid cell accumulation in breast cancer models (Welte et al., 2016). These studies demonstrate that loss of *Tp53* in cancer cells, either alone or combined with additional genetic events, leads to an infiltrative myeloid microenvironment coinciding with tumor progression. Other genetic driver mutations might influence the neutrophilic immune landscape, such as oncogenic *Kras* mutations that enhance IL-8 production (Hamarsheh et al., 2020; Sparmann and Bar-Sagi, 2004). However, in comparing each study, neutrophil phenotypes were highly diverse, underscoring the regulatory relationship between tumor genotypes and neutrophil states in cancer.

#### Tumor regulation of neutrophil quality

One of the first preclinical studies to discriminate between neutrophil states in cancer described N1/anti-tumorigenic and N2/pro-tumorigenic neutrophils within tumors, whereby transition to N2 was regulated by TGF-β (Fridlender et al., 2009). TGF-β acts in part through its ability to regulate pro-inflammatory cytokines and neutrophil chemoattractants, such as CXCR2 ligands CXCL1/2/5 (Fridlender et al., 2009; Haider et al., 2019; Yang et al., 2008), and may even synergize with CSFs in BM (Celada and Maki, 1992; Hestdal et al., 1993; Keller et al., 1991). Although TGF-β can be supplied by the tumor itself, it can also be derived from microenvironmental sources, including myeloid cells (Yang et al., 2008). Subsequent studies ascribed the terms high-density neutrophils (also known as “normal density”) or low-density neutrophils to anti/pro-tumoral subsets, based on buoyant density, granularity, and maturation state (Hsu et al., 2019; Sagiv et al., 2015). These density fractions were first described in early work in autoimmunity (Hacbarth and Kajdacsy-Balla, 1986; Pember et al., 1983; Pember and Kinkade, 1983) and later refined using mass cytometry (cytometry time of flight [CyTOF]) to identify subpopulations within each fraction associated with cancer outcomes (Shaul et al., 2020; Zhu et al., 2020). Like N2 neutrophils, the low-density neutrophil subset is driven by TGF-β and exhibits immunosuppressive properties in cancer (Sagiv et al., 2015), and is associated with advanced disease (Brandau et al., 2011; Schmielau and Finn, 2001; Shaul et al., 2020). These early discoveries were among the first to challenge our understanding of neutrophil plasticity and heterogeneity in cancer.

Since then, many studies have identified unique neutrophil phenotypes in cancer. Single-cell RNA sequencing (RNA-seq) of patient lung tumors identified five neutrophil clusters that only partially overlap with those in blood (Zilionis et al., 2019). Of these, three clusters are conserved in mice, including one that is uniquely found in tumors and not healthy tissue, with high levels of *Ctsb* and *Ccl3* (termed N5; see Fig. 2 A; Siwicki and Pittet, 2021; Zilionis et al., 2019). Similar observations were made with CyTOF on blood samples from melanoma and lung cancer patients (Shaul et al., 2020; Zhu et al., 2020), where at least five neutrophil states were identified (Zhu et al., 2020). Specific neutrophil states are associated with patient outcomes (Zhu et al., 2020; Zilionis et al., 2019); for example, N5 neutrophils (detected by immunostaining for peptidase inhibitor 3) are associated with tumor growth and worse prognosis in lung cancer patients (Zilionis et al., 2019), and partially resemble pro-tumorigenic SiglecF^hi^ neutrophils in mice (Engblom et al., 2017). Moreover, distinct functional proficiencies can be ascribed to each state, including differential capacities for phagocytosis and ROS production (Zhu et al., 2020). However, studies exhaustively testing additional functions, such as NETosis, have been limited, as most research is focused on the bulk analysis of neutrophils. As single-cell technologies have gained considerable momentum in recent years, expanding our mechanistic understanding of how distinct neutrophil states may yield unique effects in cancer immunology will be important for developing neutrophil-targeted immunotherapies. Moreover, it will be critical to understand the contribution of distinct neutrophil states to immunotherapy-related adverse events (Siwicki et al., 2021), which often necessitate discontinuation of these therapies.

ROS production by neutrophils has several functional consequences, one of which is NETosis. In laboratory models, neutrophils from mice with leukemia, breast cancer, or lung cancer are prone to NETosis compared with those from healthy mice (Demers et al., 2012). Similar observations have been reported in humans; in esophageal, gastric, and lung cancer patients, NETs are elevated in blood compared with healthy individuals (Rayes et al., 2019). Although these observations are associative, emerging work is now addressing the possibility that tumors play a causative role in NETosis. Moreover, it is becoming clear that NETs that form in response to malignancy have multifaceted effects. Early studies have shown that cancer-induced NETs act largely within the circulation, where they facilitate cancer-associated thrombosis (Demers et al., 2012; Hell et al., 2016; Thalin et al., 2016; Thomas et al., 2015) and sequester circulating tumor cells to escort metastases (Cools-Lartigue et al., 2013). Subsequent studies revealed that NETs affect essentially every step of the metastatic cascade, including primary tumor progression (Guglietta et al., 2016), invasion and migration (Park et al., 2016), survival in the circulation (Spiegel et al., 2016; Szczerba et al., 2019; Teijeira et al., 2020), chemotaxis to secondary niches (Yang et al., 2020), extravasation (McDowell et al., 2021; Spiegel et al., 2016), metastatic colonization (Wculek and Malanchi, 2015; Yang et al., 2020), and outgrowth of metastatic tumor cells (Albrengues et al., 2018; Xiao et al., 2021). However, a remaining knowledge gap is how tumors trigger NETosis—Is this a direct consequence of tumor-derived factors? Is it a systemic response to cancer-associated inflammation? Or is it a broader shift in neutrophil developmental and aging programs? Co-culture experiments with tumor cells and NETosing neutrophils have hinted toward tumor-supplied factors, such as G-CSF, IL-8, or cathepsin C (Alfaro et al., 2016; Demers et al., 2012; Demers et al., 2016; Park et al., 2016; Xiao et al., 2021), but deeper mechanistic insights are needed to dissect contributions from developmental and diurnal neutrophil states. To date, there are no clinical trials testing NET inhibitors in cancer patients; however, clinical trials with recombinant human DNase1 are being conducted in patients with COVID-19 (NCT04409925, NCT04359654, NCT04445285, NCT04541979, NCT04432987, and NCT04402944), which will provide a foundation for translating NET-targeted therapies to immuno-oncology.

Neutrophils can adopt immunosuppressive functions both systemically and within the tumor microenvironment. This is partially due to metabolic reprogramming of neutrophils in the context of cancer, where nutrient sharing between cells and tissues becomes subverted to accommodate the high energy demands of a rapidly growing tumor. The prevailing dogma is that neutrophils are almost exclusively glycolytic, and while this is true in some cancer settings (Ancey et al., 2021; Patel et al., 2018), it is not always the case. In the 4T1 transplantable mammary tumor model, the glucose-restricted microenvironment causes neutrophils to utilize mitochondrial fatty acid oxidation, leading to enhanced ROS production, T cell suppression, NETosis, and liver metastasis (Hsu et al., 2019; Rice et al., 2018). Moreover, in the spontaneous MMTV-PyMT breast cancer model, neutrophils secrete leukotrienes, which are lipid products of Alox5-mediated oxidation of arachidonic acid, to promote tumor survival and colonization within the pre-metastatic niche (Wculek and Malanchi, 2015). Changes in lipid metabolism in neutrophils have also been observed in mouse models of lung cancer, colon cancer, and rhabdomyosarcoma (Al-Khami et al., 2017; Hossain et al., 2015; Kaczanowska et al., 2021; Tavazoie et al., 2018), and in patients in association with immunosuppression (Condamine et al., 2016). Lipid uptake by neutrophils has been reported in several cancer models via fatty acid transport protein 2 (FATP2) or adipose triglyceride lipase (Li et al., 2020b; Veglia et al., 2019), which not only causes immunosuppression of antigen-specific CD8^+^ T cells (Veglia et al., 2019), but also creates an energy reservoir for metastasizing cells (Li et al., 2020b). Lipid uptake can also be regulated by tumor-derived G/GM-CSF (Al-Khami et al., 2017). Elegant models using a cell-penetrant fluorescent labeling system to study cellular neighbors of metastatic breast cancer cells in the lung microenvironment have shown that neutrophils within the immediate niche of the tumor exhibit elevated oxidative phosphorylation and ROS production (Ombrato et al., 2019), although immunosuppressive functions in this context have not been evaluated. Of note, the immunosuppressive consequences of low glucose levels in the tumor microenvironment are not restricted to granulocytes and may extend to regulatory T cells, dendritic cells, and M2-like macrophages (Angelin et al., 2017; Cubillos-Ruiz et al., 2015; Vats et al., 2006; Vitale et al., 2019; Wang et al., 2020a; Watson et al., 2021).

### Neutrophil phenotypes driven by the host environment

#### Physiologic states that influence neutrophil biology

**Figure 1.**
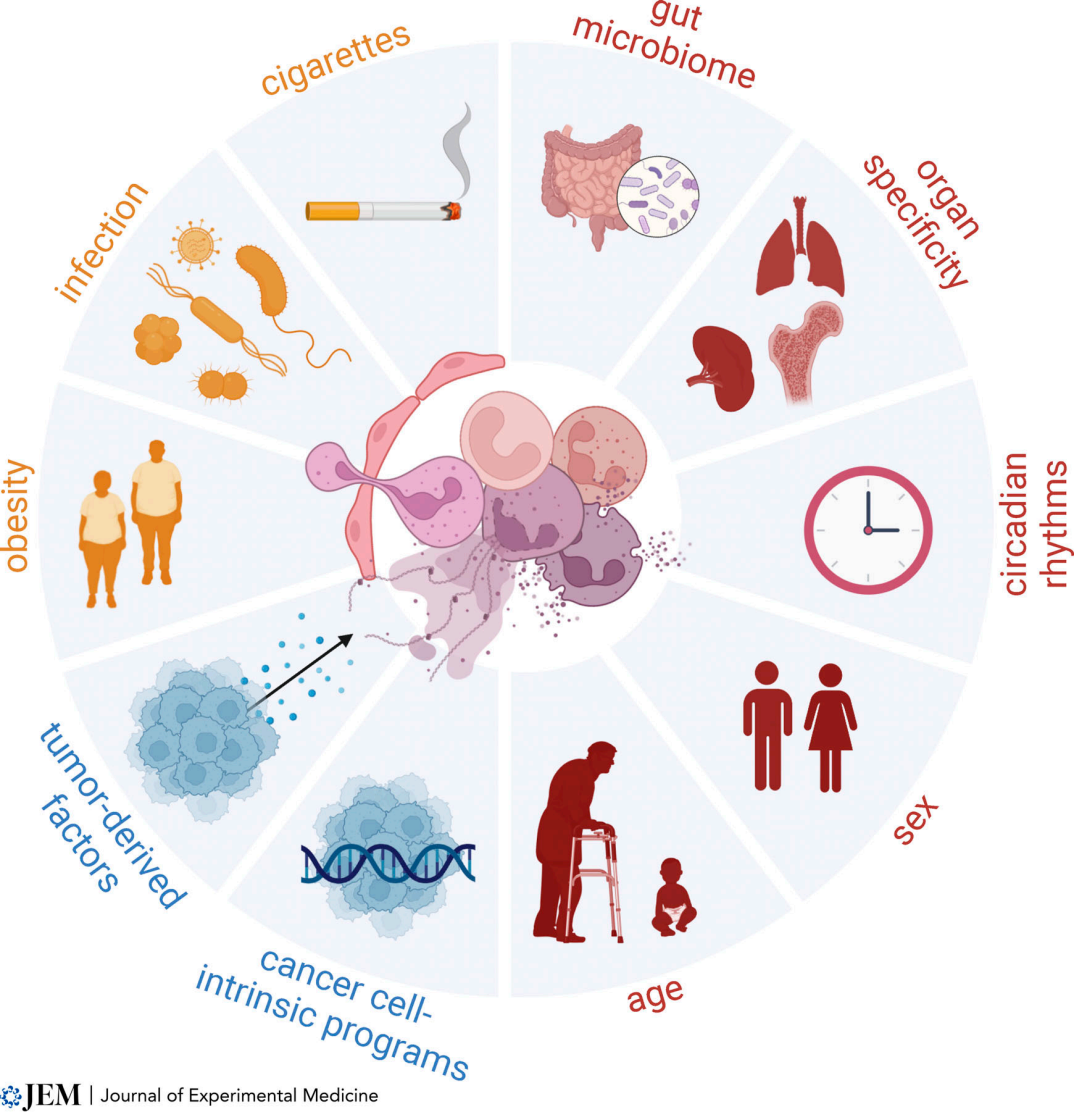
**Physiologic and pathologic states that influence neutrophil heterogeneity in cancer.** Tumor-derived factors (e.g., G-CSF, GM-CSF, CXCR2 ligands, TGF-β) and tumor genetics (e.g., *Tp53* loss, oncogenic *Kras*) regulate neutrophil recruitment and activation states in cancer. This is compounded by physiologic (e.g., age, sex, time, tissue, microbes) and pathologic (e.g., obesity, infection, cigarette smoke) states of the host that differentially prime neutrophils to respond to tumor-derived cues. Each of these factors culminate to yield a myriad of different neutrophils “flavors” in cancer that regulate essentially all steps of disease progression, from the primary site to the metastatic niche. Created with BioRender.com.

Neutrophil biology is strongly influenced by variables in host physiology, such as sex, age, circadian rhythms, and anatomical location (Fig. 1). For example, peripheral neutrophils with an immature phenotype are elevated in young men compared with young women and exhibit increased mitochondrial metabolism (Blazkova et al., 2017; Gupta et al., 2020). In contrast, women have more mature neutrophils with a heightened capacity for activation, including hyperresponsiveness to type I IFNs and enhanced capacity for NETosis (Blazkova et al., 2017; Gupta et al., 2020). Of note, ex vivo NETosis assays with mouse neutrophils have reported the opposite effect (Lu et al., 2021). Additionally, neutrophil phenotypes are not static; sex dimorphism is lost with aging, and in females, the immature neutrophil phenotype is enhanced during pregnancy as estrogen levels rise (Blazkova et al., 2017; Lu et al., 2021). Interestingly, estrogen signaling can promote intratumoral immunosuppressive activity of estrogen receptor (ER)–positive Ly6C^lo^Ly6G^+^ myeloid cells and enhance cancer progression in mice, even in ER-negative tumors (Svoronos et al., 2017). At the molecular level, multi-omics analyses of male versus female BM neutrophils in mice have confirmed sex dimorphic differences: transcriptomics revealed a female bias for extracellular matrix and cell surface–related pathways and a male bias for chromatin and cell cycle–related pathways; metabolomics showed differences in nucleotide and amino acid metabolism; and lipidomics showed a male bias for increased lipid storage (Lu et al., 2021). These fundamental differences are likely to mechanistically underlie sex dimorphic functional variations observed in steady-state, autoimmunity, and infection (Klein and Flanagan, 2016; Kourtis et al., 2014); however, the impact on tumor biology remains unexplored.

Although neutrophils are highly sex dimorphic throughout life, the effects of aging on neutrophils appear to be similar, regardless of sex (Lu et al., 2021). Aging is associated with chronic, low-grade inflammation leading to a gradual decline in immune function over time, which likely underlies the higher incidence of infection, autoimmunity, and cancer in the elderly. In mouse models, hematopoietic stem cells (HSCs) in BM are skewed toward myeloid lineage specification both in steady-state and following transplantation of aged HSCs into young mice (Rossi et al., 2005), suggesting that the age-related myeloid bias is cell intrinsic. Similar observations have been made in humans by comparing elderly (65–85 yr) and young (20–31 yr) individuals (Pang et al., 2011). In mice, analysis of male and female BM neutrophils from young (4–5 mo) and old (20–21 mo) mice has shown that aging is associated with significant changes to neutrophil gene expression, with relatively minimal changes to metabolomic or lipidomic profiles (Lu et al., 2021). Aging downregulates pathways related to chromatin organization, despite no functional differences in cell-cycle phase distribution, suggesting possible relevance to NETosis. Pathways related to autophagy, which are critical for normal neutrophil differentiation and function (Riffelmacher et al., 2017), are also upregulated with aging (Lopez-Otin et al., 2013; Lu et al., 2021). Within some lymphoid organs, including BM, LNs, and spleen, neutrophil frequencies increase in aged mice (22–24 mo; equivalent to 60–70 human yr) compared to young mice (2–3 mo; equivalent to 18 human yr), yet become functionally dysregulated (e.g., display reduced phagocytosis, increased senescence, and dysregulated NETosis; Hazeldine et al., 2014; Lu et al., 2021; Tomay et al., 2018; Wenisch et al., 2000). It remains unclear how phenotypes reported in young mice (which are typically used in cancer research) compare with those in aged mice where neutrophil function may be intrinsically different. It is possible that this variable contributes to the differences observed between neutrophils in mouse models and humans (discussed in Neutrophils with an anti-tumorigenic…; Eruslanov et al., 2017).

In both males and females, neutrophils are replenished diurnally, and the circadian regulation of neutrophil aging translates to distinct phenotypes at different times of the day (Adrover et al., 2019; Casanova-Acebes et al., 2018; Casanova-Acebes et al., 2013). In mice, neutrophils newly released into the circulation (“fresh” neutrophils) exhibit enhanced migratory programs including an enhanced capacity for vascular rolling, adhesion, and extravasation into inflamed tissues, whereas neutrophils toward the end of their lifecycle (“aged” neutrophils) show enhanced antimicrobial activity and can be retained within healthy tissues during periods of organismal activity (note that for mice, the active period is at night; Adrover et al., 2019; Zhang et al., 2015). In steady-state, circulating neutrophils “disarm” their cytotoxic functions as they age by progressively degranulating; this phenomenon likely makes them less harmful once they reach tissues and may help offset potential collateral damage to highly vascularized niches, including lung (Adrover et al., 2020). These changes are dependent on CXCR2 (Adrover et al., 2020), which, in addition to its functions above, is a master regulator of neutrophil diurnal activation (Adrover et al., 2019). A functional consequence of these time-dependent properties is that metastatic events may be diurnally regulated. Elegant circadian experiments have shown that B16F1 melanoma cells injected intravenously into syngeneic mice in the morning form overt lung metastases while injections in the evening yield minimal metastatic disease, and this difference is mitigated when neutrophils are depleted (Casanova-Acebes et al., 2018). These findings raise the possibility that the efficacy of neutrophil-targeted therapies may be different depending on the time of administration, and may even extend to other therapies that are influenced by neutrophil function. This concept was recently explored in the context of immune checkpoint blockade in melanoma patients, where it was suggested that daytime infusions may be more effective than evening infusions (Qian et al., 2021).

Neutrophil aging is regulated in part by intrinsic factors, such as the molecular clock transcription factor *Bmal1* (Adrover et al., 2019), and also extrinsic factors, such as the microbiome, which neutrophils can sense through signaling from their pattern recognition receptors (Zhang et al., 2015). The microbiome regulates neutrophil differentiation and function during infection (Balmer et al., 2014; Clarke et al., 2010; Deshmukh et al., 2014; Khosravi et al., 2014). Germ-free and antibiotic-treated mice have reduced frequencies of circulating aged neutrophils, whereas this phenomenon is reversed by fecal transplantation or LPS administration (Zhang et al., 2015). These studies suggest that dysbiosis, obesity, antibiotic use, or other factors that modify the microbiome may alter neutrophil activity both in steady-state and in response to inflammatory stimuli, including cancer. A role for the gut microbiome in regulating cancer progression and response to immunotherapy is also emerging in humans (Gopalakrishnan et al., 2018; Helmink et al., 2019; Matson et al., 2018; Routy et al., 2018). For instance, two-phase I/II clinical trials found that fecal microbiota transplantation can sensitize previously refractory cancer patients to immune checkpoint inhibitors (Baruch et al., 2021; Davar et al., 2021). These responses were associated with improved T cell infiltration and activation within tumors, and it is likely that innate immune components, including neutrophils, are also involved, given their ability to directly sense bacteria.

Neutrophils patrol healthy tissues in steady-state, and there is tissue specificity to the dynamics of neutrophil infiltration and phenotype. At homeostatic baseline, neutrophils are abundant in BM, spleen, and lung, and with lower frequencies in liver, intestine, muscle, skin, and white adipose tissue (Casanova-Acebes et al., 2018). Studies have also identified neutrophils that recirculate through LNs where they protect against infection within the lymphatics (Bogoslowski et al., 2020). Parabiosis experiments with CD45.1 and CD45.2 mice showed that blood-derived neutrophils accumulate and are retained in most tissues in the evening, whereas the intestine and liver show minimal rhythmicity (Casanova-Acebes et al., 2018). Neutrophil spatial patterning is also tissue specific, where distribution within tissues appears to be somewhat random in most organs, whereas the intestine and spleen exhibit a more purposeful localization pattern (Casanova-Acebes et al., 2018; Puga et al., 2011). Functionally, neutrophil half-life is dramatically distinct between tissues; the total lifespan ranges from 28.9 h in liver (8.7 h half-life) to 67.1 h in BM (20.2 h half-life), with blood, spleen, lung, intestine, and skin ranging in between (Ballesteros et al., 2020). Similarly in humans, a lifespan of up to 5.4 d has been reported (Pillay et al., 2010), though this remains controversial (Tofts et al., 2011). Moreover, tissue-specific analyses of RNA, protein, and chromatin have revealed striking heterogeneity between mouse neutrophils from distinct anatomical sites, including BM, lung, intestine, skin, spleen, and blood (Ballesteros et al., 2020; Xie et al., 2020). Together these findings strongly challenge the prevailing dogma that neutrophils are functionally uniform cells with rapid turnover. Despite neutrophils being the most abundant myeloid cell type in the body, a comparison of how tissue-specific properties of neutrophils might differentially impact cancers arising in different organs has not been explored.

#### Pathologic states that influence neutrophil biology

Pathologic conditions add further complexity to the influence of host physiology on immune function (Fig. 1). One condition with high relevance to oncology is infection, given the relationship between postoperative infection and poor outcomes (Scaife et al., 2013). Surgical stress, itself even in the absence of infection, is sufficient to trigger NETosis in liver ischemia-reperfusion models, leading to accelerated metastatic progression (Tohme et al., 2016). In a mouse model of intra-abdominal sepsis by cecal ligation and puncture, it has been shown that neutrophils exhibit enhanced NETosis, which facilitates trapping of tumor cells in blood and aids in the establishment of hepatic metastases (Cools-Lartigue et al., 2013). Interactions between NETs and circulating tumor cells were achieved through the expression of β1-integrins on tumor cells, which are upregulated in response to infection (Najmeh et al., 2017). Infection-enhanced metastasis can be mitigated by treatment with DNase1 in vivo, which degrades extracellular DNA to prevent NETs from escorting tumor cells into the metastatic niche (Najmeh et al., 2017). Additional studies have shown that inflammation following treatment with bacterial LPS promotes neutrophil-mediated pulmonary metastasis (Albrengues et al., 2018; El Rayes et al., 2015). This occurs in part by triggering NETosis, which enables dormant metastatic cells to re-engage their proliferative capacity (Albrengues et al., 2018) or by enhanced degranulation, resulting in secretion of proteases, such as elastase and cathepsin-G, that cleave anti-tumorigenic thrombospondin-1 (Tsp-1; El Rayes et al., 2015). Given the diversity of neutrophil states, an open question is whether neutrophils uniformly respond to infection. Single-cell RNA-seq has shown that although bacterial infection primes neutrophils for activation at the transcriptional level, it does not affect core gene signatures that distinguish subpopulation identities (Xie et al., 2020). However, it is unclear which subpopulation of neutrophils is primarily responsible for cancer-associated NETosis and how this may change during infection. Outside the context of cancer, it is established that bacterial infection and sepsis promote NETosis (Barnes et al., 2020; Brinkmann et al., 2004; Fuchs et al., 2007; Ode et al., 2018; Pilsczek et al., 2010; Qiang et al., 2013; Yipp et al., 2012); for example, in mouse models of hemorrhagic and septic shock, extracellular cold-inducible RNA-binding protein, an endogenous damage-associated molecular pattern, stimulates a unique subset of ICAM1^+^ neutrophils to exhibit enhanced NETosis and reverse-transmigration (Chen et al., 2021; de Oliveira et al., 2016; Jin et al., 2019; Murao et al., 2020; Takizawa et al., 2021). Whether ICAM1^+^ neutrophils play a specialized role during cancer metastasis by virtue of their inflammatory properties remains unknown.

Cigarette smoke is another external stimulus that has systemic effects on host immune responses and is the leading preventable risk factor for cancer mortality, accounting for ∼30% of all cancer deaths. In addition to direct genotoxic effects of cigarettes on the lung epithelium, there are dramatic effects on the lung immune landscape. Nicotine promotes the recruitment of N2-like neutrophils to the pre-metastatic lung, where they support mesenchymal-to-epithelial transition of incoming tumor cells, thereby facilitating colonization (Tyagi et al., 2021). Exposing mice to nicotine or tobacco also causes neutrophils to undergo NETosis (Albrengues et al., 2018; Hosseinzadeh et al., 2016). Proteases within NETs, including neutrophil elastase and MMP9, subsequently cleave laminin within the extracellular matrix to facilitate integrin signaling and proliferation of dormant cancer cells within the lung (Albrengues et al., 2018). Genotoxic properties of additional components of cigarette smoke, such as urethane, may also be amplified by neutrophils. Urethane directly induces neutrophil ROS, which exacerbates DNA damage and proliferation of the lung epithelium (Wculek et al., 2020). As a consequence, urethane-induced lung tumorigenesis is blunted in neutropenic *Gcsf*^*−/−*^ mice, and remarkably, when neutrophils are transiently replenished with recombinant G-CSF treatment during urethane exposure (∼1 wk), this is sufficient to rescue lung tumorigenesis up to 4 mo later (Wculek et al., 2020). These findings implicate neutrophils in tumor initiation in smokers’ lungs and corroborate previous work showing a role for neutrophils during neoplastic transformation in other tissues (Antonio et al., 2015; Katoh et al., 2013).

Finally, obesity is another major contender for the top modifiable risk factor for cancer incidence and mortality, estimated to be responsible for up to ∼20% of all cancer deaths (Calle et al., 2003; Lauby-Secretan et al., 2016; Petrelli et al., 2021). The adipose tissue microenvironment undergoes widespread immunological remodeling during weight gain, which regulates systemic inflammatory changes that contribute to metabolic syndrome (Brestoff and Artis, 2015; Hildreth et al., 2021; Jaitin et al., 2019; Vijay et al., 2020). In the non-tumor bearing setting, obesity stimulates myelopoiesis to yield elevated neutrophils and Gr1^+^ cells in multiple organs (Nagareddy et al., 2014; Xia et al., 2011). Lung neutrophils from obese and lean mice have a highly divergent transcriptome, with a significant enrichment in pathways related to oxidative stress in obese hosts coinciding with enhanced NETosis (McDowell et al., 2021). In cancer, both genetic- and diet-induced obesity models have elevated peripheral and pulmonary neutrophils that promote breast cancer metastasis to the lung in a GM-CSF–dependent manner (McDowell et al., 2021; Quail et al., 2017). Similar findings have been reported in murine models of high fat diet–induced hypercholesterolemia, where the cholesterol metabolite 27-hydroxycholesterol supports neutrophil recruitment to distal sites to promote metastasis (Baek et al., 2021; Baek et al., 2017; Ma et al., 2020). Consistently, it has been shown that a high-fat diet promotes the accumulation of Gr1^+^ myeloid cells within multiple tissues concomitant with suppressed CD8^+^ T cells and enhanced cancer progression (Clements et al., 2018; Ringel et al., 2020; Xia et al., 2011). In a liver cancer model driven by non-alcoholic fatty liver disease/non-alcoholic steatohepatitis, live imaging studies have shown that a high-fat diet promotes neutrophil infiltration into the liver, and that treatment with the anti-diabetic drug metformin is sufficient to reverse this effect and reduce early cancer progression (de Oliveira et al., 2019). Human studies have similarly found that obesity is associated with peripheral neutrophilia (Herishanu et al., 2006) concomitant with elevated markers of neutrophil activation such as myeloperoxidase and neutrophil elastase (Ali et al., 2018; Xu et al., 2015). Importantly, obesity interventions, such as bariatric surgery (Adams et al., 2007; Sjostrom et al., 2007), exercise (Moore et al., 2016), or diet (Toledo et al., 2015), have been linked to reduced cancer incidence and/or mortality in association with decreased circulating inflammatory markers.

Taken together, many host conditions influence neutrophil heterogeneity and function—this includes infection, smoking, and obesity as discussed, but also extends to other chronic inflammatory conditions (Mistry et al., 2019; Wang et al., 2020b), seasonal viral infections (Jenne and Kubes, 2015; Tate et al., 2009; Toussaint et al., 2017), the gut microbiome (Zhang and Frenette, 2019), severe COVID-19 (Barnes et al., 2020; Wilk et al., 2020; Zuo et al., 2020a), and stress (Ince et al., 2018), among other factors. The combination of pathogens and inflammatory stimuli we are exposed to throughout life is unique between individuals and contributes to trained immunity. Moreover, the effects of cancer genotypes compounded with diverse host conditions and environmental exposures underlie highly complex neutrophil heterogeneity, as has been observed by many groups. The role that each of these different factors plays in tumor progression is a growing area of investigation.

### Neutrophils with an anti-tumorigenic phenotype: An emerging paradox

It has been paradoxically observed that tumor-associated neutrophils can be protective against cancer. In some cases, tumor cells succumb to neutrophil cytotoxicity and are thus effectively cleared. Neutrophil tumoricidal functions can be blunted by catalase (Granot et al., 2011), suggesting a role for H_2_O_2_. Consistently, elevated expression of the H_2_O_2_-dependent calcium channel, transient receptor potential cation channel-M2, sensitizes metastatic tumor cells to neutrophil cytotoxicity, while sparing cells at the primary tumor site (Gershkovitz et al., 2018a). The discrepancy in susceptibility to neutrophil cytotoxicity in primary versus secondary tumors can be explained in part by cellular functions required for dissemination; metastatic tumor cells become susceptible to neutrophil cytotoxicity following epithelial-to-mesenchymal transition (Gershkovitz et al., 2018b), a process regulated by TGF-β signaling, echoing earlier studies implicating this pathway in neutrophil functional diversity (Fridlender et al., 2009; Sagiv et al., 2015). Beyond oxidative stress, neutrophils utilize additional ammunition to kill tumor cells, including granule enzymes such as elastase (Cui et al., 2021) or cathepsin-G (Sionov et al., 2019). In addition, neutrophils can kill antibody-opsonized tumor cells via trogocytosis (Bouti et al., 2021; Martinez Sanz et al., 2021; Martínez-Sanz et al., 2021; Matlung et al., 2018), which could translate to tumor-targeting antibody therapeutics. These studies suggest that harnessing neutrophil tumoricidal functions, including innate immune checkpoints (Matlung et al., 2017), may help combat cancer. However, they also exemplify that some neutrophil-derived mediators, such as elastase or ROS, can elicit both pro- and anti-tumoral effects depending on context—a concept that is still a puzzle in the field.

Complementing cytotoxicity, neutrophils can conspire with the adaptive immune system to facilitate tumor cell recognition and clearance. Co-culture experiments using cells from early-stage human lung cancer have shown that tumor-associated neutrophils enhance T cell activation compared to their blood-borne counterparts (Eruslanov et al., 2014). In turn, activated T cells prolong the lifespan of neutrophils in vitro and upregulate co-stimulatory molecules on the neutrophil plasma membrane, leading to a positive-feedback loop that perpetuates T cell stimulation (Eruslanov et al., 2014). In subsequent work, a specialized subset of tumor-associated HLA-DR^+^ neutrophils was identified in early-stage tumors, which is capable of cross-presenting exogenous tumor antigens to CD8^+^ T cells to stimulate tumor-specific effector T cell responses (Singhal et al., 2016). Others have similarly reported that FcɣR engagement converts neutrophils into antigen presenting cells that cross-present to CD8^+^ T cells to induce anti-tumor immunity in melanoma models (Mysore et al., 2021). However, over time, the ability of tumor-associated neutrophils to engage adaptive immune pathways declines as tumors progress (Singhal et al., 2016). These data support a model where neutrophils undergo an immunogenic “switch” from anti-tumorigenic to pro-tumorigenic as cancer advances; however, this temporal response may be context specific. For example, in mouse models of melanoma, neutrophils maintain some anti-tumorigenic properties in the advanced disease under specific therapeutic settings. Combining a triple therapy of chemotherapy, infusion of CD4^+^ T cells specific to the melanoma antigen Trp1, and co-stimulation or immune checkpoint blockade is sufficient to eliminate tumors, due to a significant infiltration of neutrophils that exhibit anti-tumor behavior (Hirschhorn-Cymerman et al., 2012; Hirschhorn-Cymerman et al., 2020). The increase in these anti-tumorigenic neutrophils was associated with an increase in cutaneous immune adverse events. In human melanoma samples, treatment with immune checkpoint inhibitors is associated with enhanced NETosis, which has been proposed as a potential mechanism to eradicate tumor antigen escape variants that arise in response to treatment selection pressure (often in concordance with immune-related adverse events; Hirschhorn-Cymerman et al., 2020). Although many studies have characterized the role of neutrophils in cancer progression, how neutrophils are altered in response to specific therapies at different stages of disease remains unanswered and needs further exploration.

Many anti-tumorigenic neutrophil functions have been described in humans, raising questions about species-specific roles in cancer (Eruslanov et al., 2017). Single-cell RNA-seq of human and mouse neutrophils from lung tumors showed considerable, but not complete, overlap between species (Zilionis et al., 2019). At the functional level, several differences have been reported. First, the NETosis potential of circulating neutrophils from mice versus patients with cancer is distinct, including their ability to release NETs in response to G-CSF (Arpinati et al., 2020). Second, the tumoricidal functions of neutrophils may be species specific, based on differences in their granule/secretory profile (Cui et al., 2021; Rausch and Moore, 1975) or utilization of distinct ROS pathways (Bagaitkar et al., 2012). Third, mouse and human neutrophils differ in their expression of Arg-1 and ability to metabolize arginine (Jacobsen et al., 2007; Munder et al., 2005; Rodriguez et al., 2007). This could affect T cell responses, as decreased arginine availability in the tumor microenvironment is associated with T cell immunosuppression. Another explanation for the protective role of neutrophils in cancer is the possibility of stage-dependent enrichment for specific neutrophil states. For example, when comparing the immune infiltrate of highly and poorly metastatic murine tumors, both increase myeloid infiltration in the pre-metastatic lung; however, poorly metastatic tumors also secrete prosaposin, which induces Tsp-1 expression in lung-infiltrating Gr1^+^ cells to impair metastasis (Catena et al., 2013; Wang et al., 2016). Similar mechanisms between early and advanced tumors in patients have yet to be compared.

### Neutrophil heterogeneity is observed throughout granulopoiesis

A major source of neutrophil heterogeneity comes from the dynamics of their maturation and release from BM. Neutrophils arise from HSCs in BM, which give rise to multipotent progenitors, then common myeloid progenitors, granulocyte–monocyte progenitors (GMPs), and neutrophil-committed precursors (Table 1). This linear model may in part underlie our confounded understanding of neutrophil heterogeneity, as neutrophil ontogeny and phenotypic plasticity are likely to be more complex. Learning from other myeloid lineages, monocyte heterogeneity and functionality reflect both ontogeny and response to microenvironmental stimuli. Inflammatory monocytes can arise independently from both GMPs and monocyte–dendritic cell progenitors (MDPs), where GMPs give rise to neutrophil-like monocytes, while MDPs give rise to monocytes that can further differentiate into dendritic cells (Yanez et al., 2017). Moreover, factors within the microenvironment confer a preference for monocyte maturation along one of these developmental trajectories; LPS favors neutrophil-like monocyte maturation from GMPs, while exposure to CpG favors the MDP lineage (Yanez et al., 2017). These developmental mechanisms may in part explain the functional breadth of monocyte and macrophage identities. Indeed, the lessons that we have learned from monocytes may be relevant to a modified understanding of granulopoiesis and neutrophil plasticity.

**Table 1.** Cell surface markers defining neutrophil developmental states

Acronym	Full name	Function	Surface markers (mouse)	Surface markers (human)	Overlapping states	References
CMP	Common myeloid progenitors	High proliferation, low self-renewal, multipotent	LKS^−^ CD34^int^ CD16/32^int^ Flt3^+^ CD115^lo^ Antibody combination excludes monocyte-DC progenitors, known as “MDP”	Lin^−^ CD34^+^ CD38^+^ CD45RA^−^		Kwok et al., 2020; Yanez et al., 2017
GMP	Granulocyte–monocyte progenitors	High proliferation, low self-renewal, oligopotent	LKS^−^ CD34^+^ CD16/32^hi^ Ly6C^−^ Antibody combination excludes committed granulocyte progenitors and monocyte progenitors	Lin^−^ CD34^+^ CD38^+^ CD45RA^+^		Yanez et al., 2017; Kwok et al., 2020
proNeu1	Pro-neutrophils (stage 1)	Committed progenitors; expand in BM during emergency granulopoiesis at the expense of monocytes	LKS^−^ CD34^+^ CD16/32^hi^ Ly6C^+^ CD115^lo^ CD81^+^ CD11b^−^ CD106^−^	CD15^+^ CD66b^+^ CD11b^+^ CD49d^hi^ SSC^lo^ (also CD34^lo^ CD38^lo^)	Mouse: G0 (Xie et al., 2020); Human: eNeP/N1 (Dinh et al., 2020)	Kwok et al., 2020; Evrard et al., 2018
proNeu2	Pro-neutrophils (stage 2)	Intermediate progeny; do not expand during emergency granulopoiesis	LKS^−^ CD34^+^ CD16/32^hi^ Ly6C^+^ CD115^lo^ CD81^+^ CD11b^+^ CD106^+^	CD15^+^ CD66b^+^ CD11b^+^ CD49d^int^ SSC^hi^ (also CD34^−^ CD38^−^)	Mouse: G1 (Xie et al., 2020); Human: N1 (Dinh et al., 2020)	Kwok et al., 2020; Evrard et al., 2018
preNeu	Neutrophil precursors	High proliferation, low motility, low effector functions; expand in BM and spleen during emergency granulopoiesis	LCS^−^ cKit^int^ Ly6C^+^ CD11b^+^ Ly6G^lo^ CXCR2^−^ CXCR4^hi^	CD15^+^ CD66b^+^ CD11b^+^ CD49d^int^ CD101^−^	Mouse: G2 (Xie et al., 2020), NeuP (Kim et al., 2017), C1/NeP (Zhu et al., 2018); Human: N2 (Dinh et al., 2020)	Kwok et al., 2020; Evrard et al., 2018
immNeu^a^	Immature neutrophils	Intermediate proliferation, motility and effector functions	LCS^−^ cKit^lo^ Ly6C^lo^ CD11b^+^ Ly6G^int^ CXCR2^−^ CXCR4^lo^	CD15^+^ CD66b^+^ CD11b^+^ CD49d^−^ CD101^+^ CD16^int^ CD10^−^	Mouse: G3 (Xie et al., 2020), C2 (Zhu et al., 2018); Human: N3 (Dinh et al., 2020)	Kwok et al., 2020; Evrard et al., 2018
mNeu^a^	Mature neutrophils	Low proliferation, high motility, high effector functions	LCS^−^ cKit^−^ Ly6C^lo^ CD11b^+^ Ly6G^hi^ CXCR2^+^ CXCR4^−^	CD15^+^ CD66b^+^ CD11b^+^ CD49d^−^ CD101^+^ CD16^hi^ CD10^+^	Mouse: G4 (BM) and G5 (blood; Xie et al., 2020); Human: N4 and 5 (Dinh et al., 2020)	Kwok et al., 2020; Evrard et al., 2018

LKS^**+**^, Lin^−^ cKit^+^ Sca1^+^; LKS^−^, Lin^−^ cKit^+^ Sca1^−^; LCS^−^, Lin^−^ CD115^−^ SiglecF^−^; Lin, cocktail of lineage marker antibodies, which should include anti-CD11b up to the GMP stage, but exclude it for analysis of proNeu-mNeu.

a CXCR2 can be downregulated within tumors; to define mouse immNeu and mNeu in this context, CD101 can be used (CD101^−^ immNeu and CD101^+^ mNeu; Evrard et al., 2018).

**Figure 2.**
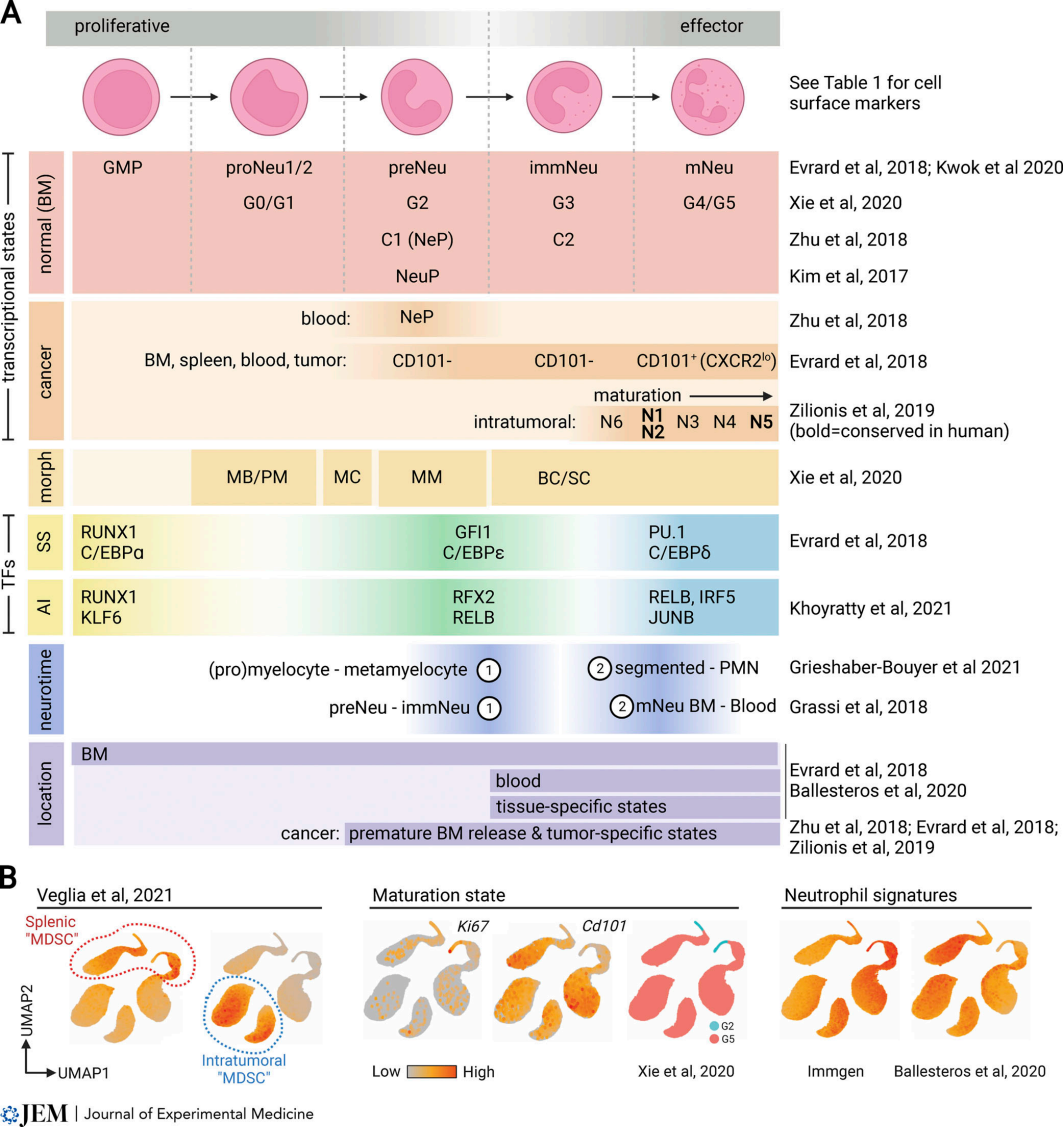
**Neutrophil maturation and activation. (A)** Comparison of neutrophil states described in landmark studies using single-cell analyses. Overlaid are transcriptional states in normal development and cancer, cell morphology (morph), transcription factor (TF) activity in steady-state (SS) and acute inflammation (AI), neutrotime developmental transition waves, and anatomical location. MB/PM, myeloblasts and promyelocytes; MC, myelocytes; MM, metamyelocytes; BC/SC, band cells and segmented neutrophils. **(B)** Projection of MDSC single-cell RNA-seq data from Veglia et al. (2021a) onto established neutrophil states (comparative datasets obtained from Xie et al. [2020], Immgen [Aran et al., 2019], and Ballesteros et al. [2020]). All intratumoral polymorphonuclear populations referred to as MDSC express canonical signatures of neutrophil maturation and identity states. Created with BioRender.com.

In recent years, single-cell technologies have made substantial advancements toward defining neutrophil maturation from GMPs (Dinh et al., 2020; Drissen et al., 2016; Evrard et al., 2018; Kwok et al., 2020; Olsson et al., 2016; Velten et al., 2017; Xie et al., 2020; Zhu et al., 2018). Across studies, several new terms have been coined for overlapping neutrophil developmental states, which are in need of consolidation (Table 1 and Fig. 2 A). In mouse BM, GMPs mature into pro-neutrophils (proNeu [Dinh et al., 2020; Evrard et al., 2018; Kwok et al., 2020; Muench et al., 2020], corresponding to clusters G0/G1 in similar work [Xie et al., 2020]) and then into highly proliferative, poorly motile precursors (preNeu) that drive expansion within spleen and BM (Evrard et al., 2018). Cells defined as preNeu (Evrard et al., 2018) are transcriptionally similar to unipotent neutrophil progenitors (NeP; Zhu et al., 2018), neutrophil precursors (NeuP; Kim et al., 2017), and to a G2 cluster that resembles myelocytes/metamyelocytes (Xie et al., 2020; also see Ng et al., 2019). Subsequently, preNeu differentiate into immature (Ly6G^int^CXCR2^−^; immNeu) or mature (Ly6G^hi^CXCR2^+^; mNeu) neutrophils that are non-proliferative and mediate trafficking and effector functions (Evrard et al., 2018). ImmNeu overlaps transcriptionally with a band cell-like G3 cluster that expresses low *Cxcr2* and high secondary granule genes (Xie et al., 2020), and with late-stage progenitors (cluster C2; high Ly6G expression), as confirmed by adoptive transfer experiments with NeP (Zhu et al., 2018). Mature neutrophils correspond to cluster G4 from Xie et al. (2020), representing the most mature state in BM with high expression of *Mmp8* and *Cxcl2*, while the G5 cluster was the most mature overall and predominant in blood (Xie et al., 2020). A summary of overlapping states can be found in Table 1.

Neutrophil development coincides with shifts in chromatin accessibility and transcription factor activity (Ai and Udalova, 2020; Ballesteros et al., 2020). Developmental trajectory analysis of single-cell RNA-seq data unveiled a developmental continuum known as “neutrotime,” which spans the maturation spectrum from neutrophil precursors in BM to mature neutrophils in blood and spleen (Grieshaber-Bouyer et al., 2021). The sharpest changes in neutrotime occur during granulopoiesis (transition from immature to mature) and mobilization (transition from BM to blood; Grieshaber-Bouyer et al., 2021). Consistently, in steady-state and inflammation, neutrophil chromatin landscapes and transcription factor networks shift in waves. In steady-state, the initiation of granulopoiesis in BM involves RUNX1 and C/EBPα transcription factors within GMPs, followed by a shift to GFI1 and C/EBPε in preNeu during early neutrophil differentiation, and then C/EBPδ and PU.1 as mNeu enter the circulation (Evrard et al., 2018). The temporal expression of C/EBP-family members mirrors the pattern of granule enzyme expression; primary granule enzymes (e.g., *Mpo*) are expressed at the GMP stage, secondary granules (e.g., *Ltf*) at the preNeu/immNeu stage, and tertiary granules (e.g., *Mmp8*) at the mNeu stage, corresponding to *Cebpa*, *Cebpe*, and *Cebpd*, respectively (Evrard et al., 2018). In comparison, chromatin profiling of neutrophils combined with genetic validation approaches has identified transcription factors involved in neutrophil maturation during acute inflammation. For example, RUNX1 and KLF6 drive neutrophil maturation in BM, and after cells are mobilized, chromatin is remodeled to enable access by RFX2 and RELB to promote survival (Khoyratty et al., 2021). Within tissues, another chromatin remodeling event enables the transcriptional activity of RELB, IRF5, and JUNB, which license innate effector functions that are pre-programmed in early developmental stages (Khoyratty et al., 2021). These findings echo those from earlier work describing two major waves of chromatin remodeling between developmental transitions from (pro)myelocyte to metamyelocytes and from segmented neutrophils to polymorphonuclear neutrophils (Grassi et al., 2018), and extend them by charting a transcriptional blueprint for each transition. The remaining knowledge gaps include understanding how environmental signals integrate with transcription factor landscapes to yield functional outputs, and how the release of neutrophils from BM at different maturation states is regulated.

Functionally, there are still many unknowns surrounding the kinetics of neutrophil maturation, trafficking, and effector function (Barros-Becker et al., 2020; Hind et al., 2021; Houseright et al., 2021; Klemm et al., 2021). For example, some neutrophils are capable of reverse transmigration from inflamed tissues back into the circulation (de Oliveira et al., 2016; Wang et al., 2017). Moreover, in cancer, multiple neutrophil developmental states co-exist: in orthotopic mouse models of pancreatic cancer, CD101^−^ immNeu accumulate intratumorally in association with disease progression (Evrard et al., 2018), corroborating studies that have reported a pro-tumorigenic role for neutrophils with a banded (immature) nuclear morphology (Coffelt et al., 2015; Hsu et al., 2019; Sagiv et al., 2015). Consistently, in orthotopic melanoma models, NeP are elevated in BM, blood, and tumor, and they promote tumor growth in association with elevated expression of PD-L1 (Zhu et al., 2018). In melanoma patients, human NeP are found at higher frequencies in blood compared to healthy donors, where they are extremely rare (Zhu et al., 2018). Even earlier developmental stages can be detected in blood and tumors of lung cancer patients, including precursors to NeP/preNeu states (Dinh et al., 2020). Moreover, extramedullary granulopoiesis has been described in the context of cancer where neutrophil maturation in spleen yields an immunosuppressive phenotype (Alshetaiwi et al., 2020; Cortez-Retamozo et al., 2012; Mastio et al., 2019). Therefore, shifts in the developmental states of granulocytes, their premature egress from BM at different stages (which may influence granule cargo), and their distinct functional contributions to tumor growth shed light on the high degree of neutrophil heterogeneity observed in cancer. This echoes findings in other inflammatory conditions, like systemic lupus erythematosus, where distinct neutrophil states identified through single-cell RNA-seq uniquely regulate disease pathogenesis (Mistry et al., 2019).

Finally, it remains unknown how trained immunity at the individual (host) level may influence this complex system. Studies in animal and plant models that lack adaptive immunity have shown that innate immune cells or their developmental precursors can become “trained” following exposure to an inflammatory stimulus, resulting in an altered response to secondary challenges even after cells have returned to a resting state (Chavakis et al., 2019; Netea et al., 2020). Trained immunity is thus different between individuals, depending on history of pathogen exposures, vaccination, cancer, or other inflammatory stimuli. Indeed, studies have identified β-glucan, Bacillus Calmette-Guérin, and other vaccines as prototypical agonists of trained immunity, mediated by education of myeloid cells within the BM and myeloid-bias progenitor expansion (Kaufmann et al., 2018; Mitroulis et al., 2018); however, trained immunity can also be achieved through sterile triggers, such as diet-induced changes to myeloid progenitor reprogramming (Christ et al., 2018). Recent studies have explored the role of trained immunity in cancer in the context of granulopoiesis. β-glucan induces myeloid expansion concomitant with elevated innate immune signaling mediators, such as IL-1β and GM-CSF (Mitroulis et al., 2018), which mediate neutrophil expansion in tumors. Treatment of mice with β-glucan 1 wk prior to subcutaneous inoculation of B16F10 melanoma or Lewis lung carcinoma lung cancer cells into syngeneic hosts blunts tumor growth, even in *Rag1*^*−/−*^ mice that lack T and B cells (Kalafati et al., 2020). Mechanistically, β-glucan causes epigenetic and transcriptomic reprogramming of granulopoiesis, yielding progenitor cells with enhanced IFN signaling and tumoricidal mature neutrophils with elevated ROS (Kalafati et al., 2020). Moreover, the anti-tumor effect of β-glucan is maintained and transferrable following BM transplantation or neutrophil adoptive transfer from β-glucan–treated donor mice into naive recipients (Kalafati et al., 2020). Outside the context of cancer, activated neutrophils can prime macrophages to exhibit long-term responses against parasitic infection (Chen et al., 2014), raising the possibility that neutrophils may participate in training of other myeloid cells in tumors. These findings support the notion that myeloid-targeted immunotherapies that aim to reprogram, rather than deplete, target cells are an attractive therapeutic approach to harnessing anti-tumor immunity.

## Discrepancies in neutrophil research and proposed solutions

Owing to the complexity of neutrophil diversity, it is not surprising that we have encountered several experimental discrepancies as a field. First, amongst many possible functional states, neutrophils can be immunosuppressive, thus challenging the designation of granulocytic myeloid-derived suppressor cells (G-MDSCs; also known as PMN-MDSC) as a distinct population. G-MDSC and neutrophils are indistinguishable by archetypal cell surface markers and have identical granular and nuclear morphologies. Moreover, there are no genetic models to trace or target G-MDSCs due to lack of genetic differences from neutrophils. Instead, antibody depletion via anti-Gr1 or anti-Ly6G is often used to test their functional role in disease; however, these antibodies also deplete neutrophils, making causative functional assessment of MDSCs uninterpretable. For associative functional studies, it has been suggested that identification of MDSCs requires confirmation of immunosuppression ex vivo (Bronte et al., 2016; Veglia et al., 2021b), but often cell surface markers are used alone. Single-cell RNA-seq studies have attempted to identify MDSC in tumors (Veglia et al., 2021a); however, putative G-MDSCs share transcriptional similarities with canonical neutrophils as defined by Immgen (Heng et al., 2008), as well as signatures of mature neutrophils (Evrard et al., 2018; Xie et al., 2020) across multiple tissues (Ballesteros et al., 2020; Fig. 2 B). Given the diversity of neutrophil identities, which can include immunoregulatory functions, it remains controversial whether G-MDSCs should simply be called “immunosuppressive neutrophils” and thus terminology is used inconsistently between groups. Although putative surface markers have been identified to distinguish G-MDSC and neutrophils such as CD84, CD14, FATP2, or LOX-1 (Alshetaiwi et al., 2020; Condamine et al., 2016; Veglia et al., 2021a; Veglia et al., 2019), these have not been used by the broader research community and they may be expressed by bona fide neutrophils under certain conditions. Given the lack of existing genetic and phenotypic markers to distinguish bona fide neutrophils from putative G-MDSC, it has been suggested that the monolithic MDSC terminology be dropped in favor of a more colorful view of myeloid biology that embraces the high degree of cellular and functional heterogeneity (Hegde et al., 2021). We support this view and urge scientists to consider the literature on both neutrophils and G-MDSCs to inform ongoing research within these evolving fields.

Second, antibody-mediated neutrophil depletion has context-dependent efficacy. The most common antibodies used are anti-Ly6G (clone 1A8, rat IgG2a) and anti-Gr1 (clone RB6-8C5, rat IgG2b); anti-Ly6G is often preferred since mature neutrophils are Ly6G^+/hi^, while Gr1 also targets monocytes, some immature myeloid cells, and even a subset of CD8^+^ memory T cells by virtue of Ly6C expression. However, in C57BL/6 mice, anti-Gr1 effectively ablates neutrophils, whereas anti-Ly6G is less effective because depletion is slower than neutrophil repopulation from BM (Boivin et al., 2020; Faget et al., 2018 *Preprint*). Despite this, numerous studies have used anti-Ly6G to deplete neutrophils successfully, suggesting that experimental design is key: first, dose and duration are variables that affect any mAb-based depletion approach. Short-term experiments (1–2 d) with anti-Ly6G result in highly effective neutrophil depletion (Deniset et al., 2017; Lee et al., 2018; Siwicki et al., 2021); however, in longer trials (>3–7 d), neutrophil numbers may begin to rebound (Deniset et al., 2017; Moses et al., 2016). Second, there are differences in neutrophil depletion efficacy depending on experimental context. Comparative analyses with anti-Gr1 and anti-Ly6G mAbs showed enhanced depletion in Balb/c and FVB/n backgrounds compared to C57BL/6, especially when C57BL/6 mice were aged (Boivin et al., 2020; Faget et al., 2018 *Preprint*). Moreover, neutrophils are more difficult to eliminate in certain tissues, such as BM (Pollenus et al., 2019). Third, for experimental contexts in which anti-Ly6G is ineffective, a more durable protocol has been developed via co-administration of rat anti-mouse Ly6G and anti-rat mAbs, which enhance the killing efficacy of anti-Ly6G to deplete neutrophils for at least 18 d in C57BL/6 models (Boivin et al., 2020; Faget et al., 2018 *Preprint*). Finally, neutrophil depletion efficacy cannot be validated using anti-Ly6G (1A8), as antigen masking produces false-negative staining (Boivin et al., 2020). Alternative strategies include intracellular Ly6G staining, histology for myeloperoxidase (MPO) or neutrophil elastase (NE), or reporter mice, such as *LysM-cre* or *Ly6G-cre*, combined with Ly6C^lo/int^, and side scatter assessment (Boivin et al., 2020; Deniset et al., 2017; Hasenberg et al., 2015). Genetic neutrophil depletion approaches, as discussed below, may also provide functional confirmation of antibody effects (Ballesteros et al., 2020).

Common genetic approaches to target or deplete neutrophils in vivo include the *Mrp8-cre* model (Passegue et al., 2004), either combined with a specific floxed allele or ROSA-DTA mice (cre-dependent expression of diphtheria toxin), respectively. The *Mrp8-cre* model targets ∼80% of mature neutrophils in vivo; however, *Mrp8* is also expressed in 10–20% of GMPs (Passegue et al., 2004). Alternatively, the *Ela2-cre* model targets mature neutrophils expressing elastase, a serine protease within neutrophil primary granules (Tkalcevic et al., 2000; Wculek and Malanchi, 2015). Newer models, such as the *Ly6G-cre* mouse (Hasenberg et al., 2015), offer a more specific alternative; however, the efficacy of recombination is purportedly allele-specific depending on the particular floxed strain being used. Further, activation of recombinase under control of this gene occurs late in neutrophil development, such that proteins already produced may persist for the short lifetime of the cell. For combinations with fluorescent reporter strains for intravital microscopy or other imaging modalities (such as the Ai9 cre reporter mouse), the *Ly6G-cre* mouse is an excellent option for neutrophil-specific tracing (Hasenberg et al., 2015). However, for gene deletion studies, this model should be used in parallel with *Mrp8-cre* or *Ela2-cre*, and recombination should be carefully validated.

Third, methods to target NETosis have raised debate within the field. Genetic knockout studies have shown that protein arginine deiminase 4 (PAD4) is required for nuclear decondensation and nuclear rupture preceding NETosis (Li et al., 2010). Yet, some studies have found that targeting PAD4 is effective against NETosis (Hemmers et al., 2011; Li et al., 2010; Martinod et al., 2013; Munzer et al., 2021; Thiam et al., 2020), while others have not (Claushuis et al., 2018; Guiducci et al., 2018; Kenny et al., 2017; Tsourouktsoglou et al., 2020). There are several plausible explanations for this discrepancy: First, some defining features of NETosis can be mimicked in other contexts. Examples include leukotoxic hypercitrullination (involving non-bactericidal hyperactivation of PAD leading to DNA extrusion), constitutively defective mitophagy (resulting in mitochondrial DNA expulsion; Caielli et al., 2016; Lood et al., 2016; Yousefi et al., 2009), or epigenetic regulation of pluripotency (whereby PAD4-mediated citrullination promotes an open chromatin structure; Christophorou et al., 2014). Thus, a standardized readout for NETosis is needed. Although citrullinated histone H3 (H3cit) is a reasonably specific marker for NETs in disease models since PAD4 deficiency/inhibition prevents citrullination of histones, histological assessment of NE or MPO associated with extracellular DNA is required to measure NETs in the context of PAD4 blockade. Second, NETosis may not always rely on PAD4, as observed in models of pneumosepsis (Claushuis et al., 2018). NETosis can be NOX2 dependent (induced by phorbol esters, LPS, etc.) or NOX2 independent (induced by calcium ionophores, UV light, etc.; Douda et al., 2015; Fuchs et al., 2007; Lood et al., 2016; Parker et al., 2012; Pilsczek et al., 2010). Hypercitrullination of histones only occurs during calcium ionophore-activated NETosis, suggesting PAD4 may contribute specifically to NOX2-independent NETosis (Douda et al., 2015; Khan and Palaniyar, 2017). Third, mouse background strain may influence PAD4 dependency. C57BL/6 mice have a Th1-type bias, whereas other strains tend to favor Th2 responses, therefore, we recommend that background strains be reported in publications. Finally, PAD4 reliance may be context dependent. For example, “aged” neutrophils exhibit lower NETosis than “fresh” neutrophils newly released from BM (Adrover et al., 2019); neutrophils responding to bacterial infection exhibit pronounced NETosis compared with those responding to sterile injury (Yipp et al., 2012); and PAD4 inhibitor efficacy may have species-specific variation (Arpinati et al., 2020; Lewis et al., 2015). Of note, it is uncertain how targeting NETs might impact innate immune responses in humans, given their role in autoimmunity (Khandpur et al., 2013; Li et al., 2020), aging (Martinod et al., 2017), emergency granulopoiesis, vascular inflammation (Knackstedt et al., 2019), and other inflammatory conditions (Jorch and Kubes, 2017; Papayannopoulos, 2018; Phillipson and Kubes, 2011). This question is being addressed for the first time in patients with severe COVID-19 who exhibit elevated NETosis (Ackermann et al., 2021; Barnes et al., 2020; Middleton et al., 2020; Zuo et al., 2020b *Preprint*), which will provide insight for cancer patients.

## Concluding remarks: Open questions in neutrophil biology and cancer

Going forward, it will be critical to reconsider how neutrophils are classified and studied in the laboratory setting. First, there is a knowledge gap in reconciling the root cause of neutrophil heterogeneity. Adopting principles from the mononuclear phagocyte system, in which cells are classified developmentally and phenotypically (Guilliams et al., 2014), may help clarify tissue-specific neutrophil biology in steady-state and inflammation. Although neutrophils originate from definitive hematopoiesis, efforts in this direction are now emerging (Evrard et al., 2018; Kim et al., 2017; Muench et al., 2020; Zhu et al., 2018), and there is a growing appreciation that functionally distinct developmental identities can co-exist in cancer. We propose to use the terms “states” to refer to phenotypically distinct neutrophil populations, including those that are immunosuppressive since it is the environment that appears to drive neutrophil heterogeneity via shifts in maturation and/or activation.

Second, there is a need to standardize techniques. This includes methods to deplete neutrophils with antibodies, discriminate between neutrophil states, and validate NET targeting approaches. Of note, currently, the preferred methods for NET detection are immunohistochemistry (association of MPO, NE, or Ly6G with extracellular DNA, or H3cit in the presence of MPO/DNA complexes) or dual-target ELISA (e.g., anti-elastase and anti-DNA-peroxidase), and new detection tools are emerging, including antibodies against histone H3 cleavage events specific to NETs in humans (Tilley et al., 2021 *Preprint*). However, alternative approaches are readily used, including H3cit ELISA/immunohistochemistry or flow cytometry for different combinations of MPO, H3cit, and/or SYTOX viability dyes, while these provide reasonable estimates, they are not conclusive. Although we see value in these techniques under certain circumstances, for example, to analyze limited patient material, we recommend that orthogonal approaches to validate findings are employed.

Third, most studies are focused on bulk analysis of neutrophils. However, single-cell technologies can resolve shifts in the relative proportions of neutrophil states that co-exist outside the BM. Using these datasets to map differentiation trajectories and epigenetic regulation of neutrophil development using single-cell assay for transposase-accessible chromatin sequencing may also reveal relationships not evident from the transcriptome. These efforts will be particularly informative to understand molecular mechanisms and transcriptional regulators that govern neutrophil maturation and function (Evrard et al., 2018; Muench et al., 2020; Weiss et al., 2015). Moreover, they will allow us to resolve nomenclature for various neutrophil developmental stages (see Table 1 and Ng et al., 2019). In addition, in vivo techniques to trace or target individual cell subsets would be of value (Harvie and Huttenlocher, 2015). For example, three neutrophil states are conserved within tumors in both humans and mice (Zilionis et al., 2019). Whether these neutrophil states are functionally plastic and/or whether they can be individually targeted has yet to be determined.

Finally, our knowledge of neutrophils in cancer largely depends on data from murine models. However, it is clear that there are some species-specific differences in neutrophil regulation of tumor biology. In human studies, most experimental approaches are limited to ex vivo analyses of blood neutrophils, transcriptomic profiling of tumor-associated neutrophils, and basic correlations with clinical outcomes. Although these approaches are informative, they shed light on disease association and not causation. Therefore, a comprehensive understanding of the functional impact of human neutrophils on cancer is lacking and needs to be further elucidated to develop relevant therapeutic strategies.

Taken together, our fundamental understanding of neutrophil maturation, heterogeneity, and function in the context of cancer has recently seen remarkable advances with emerging fate-tracing, high-parameter, and single-cell technologies that are now allowing us to study fundamental neutrophil biology at unprecedented depth. The next challenge is to reconcile existing data and unify nomenclature so that our collective discoveries can be integrated and directly compared. Ultimately, we aim to harness our emerging understanding of neutrophil phenotypic heterogeneity to characterize neutrophil states at the functional level, and effectively exploit them for therapeutic purposes.
